# Age-Associated Trends in Diagnosis and Prevalence of Infection with HIV Among Men Who Have Sex with Men — United States, 2008–2016

**DOI:** 10.15585/mmwr.mm6737a2

**Published:** 2018-09-21

**Authors:** Andrew Mitsch, Sonia Singh, Jianmin Li, Alexandra Balaji, Laurie Linley, Richard Selik

**Affiliations:** 1 Division of HIV/AIDS Prevention, National Center for HIV/AIDS, Viral Hepatitis, STD, and TB Prevention, CDC.

In 2016, two thirds of diagnosed human immunodeficiency virus (HIV) infections in the United States were attributed to male-to-male sexual contact (*1*). The risk for sexual acquisition and transmission of HIV changes through the lifespan (*2*); to better guide prevention efforts for gay, bisexual, and other men who have sex with men (MSM*), CDC analyzed National HIV Surveillance System^†^ (NHSS) data for MSM aged ≥13 years by age group (13–29, 30–49, and ≥50 years) in 50 states and the District of Columbia (DC). During 2008–2016, the annual number of diagnoses of HIV infection increased 3% per year among MSM aged 13–29 years, decreased 4% per year among those aged 30–49 years and was stable for MSM aged ≥50 years. The number of HIV diagnoses among MSM aged 13–29 years was four times that of MSM aged ≥50 years. During 2008–2015, the number of MSM aged ≥50 years living with diagnosed HIV infection (prevalence of HIV infection) increased an average of 11% per year and at year-end 2015 was three times that of MSM aged 13–29 years. Racial/ethnic disparities in HIV infection persisted, particularly among younger black/African American MSM who accounted for 49% of all diagnoses among MSM aged 13–29 years during 2008–2016. To avert the most infections and improve health outcomes (*3*), sexually active MSM at risk for HIV infection should be tested at least once a year, and, if positive, linked to and retained in HIV medical care to achieve viral suppression (*4*). Those testing negative should be provided HIV prevention services, including preexposure prophylaxis (PrEP) (*5*).

All states and U.S. dependent areas report cases of HIV infection and associated patient demographic and clinical information to NHSS. CDC analyzed data reported through December 2017 from the U.S. states and DC, statistically adjusted for missing risk factor information (*6*), for MSM aged ≥13 years. Data were analyzed for MSM aged 13–29, 30–49, and ≥50 years.

Trends in annual diagnoses of HIV infection among MSM during 2008–2016 were measured using estimated annual percent change (APC) tabulated by age group and race/ethnicity and by age group and region of residence at diagnosis. The APC is calculated by using a generalized log linear model. Prevalence trends among MSM living with diagnosed HIV infection were measured using APCs tabulated by age group and last known jurisdiction of residence at year-end during 2008–2015. Changes were considered statistically significant if the APC’s 95% confidence interval (CI) excluded zero.

Among 236,150 MSM with HIV infection diagnosed during 2008–2016, a total of 106,258 (45%) were aged 13–29 years, 100,857 (43%) were aged 30–49 years, and 29,034 (12%) were aged ≥50 years (Table 1). During this period, the annual number of diagnoses increased among MSM aged 13–29 years (APC = 2.9). The largest percentage increases in HIV diagnoses in this age group were among American Indians/Alaska Natives (APC = 14.8), Asians (12.0), and residents of the South (3.7). Among MSM aged 30–49 years, the annual number of diagnoses decreased (APC = -3.5). Among those aged ≥50 years, the overall trend was stable, although diagnoses increased among Asians (APC = 7.0) and Hispanics/Latinos (4.1). During 2008–2016, among MSM aged 13–29 years, blacks/African Americans (blacks) accounted for 49%, Hispanics/Latinos for 25%, and whites for 19% of diagnoses of HIV infection; among MSM aged 30–49 years, blacks and Hispanic/Latinos each accounted for 28% of diagnoses; and among MSM aged ≥50 years, blacks accounted for 25% of diagnoses.

**TABLE 1:** Trends in annual numbers of diagnoses of HIV infection among men who have sex with men* aged ≥13 years, by age group and race/ethnicity and by age group and region of residence at diagnosis — National HIV Surveillance System, United States and District of Columbia, 2008—2016

Characteristic		Year of diagnosis				
		2008	2016	2008–2016		
Age at diagnosis	Characteristic	No. (%)	No. (%)	No. (%)	Estimated APC	
					% (95% CI)	P-value^†^
13–29 yrs	**Race/Ethnicity**					
	AI/AN	27 (0.3)	69 (0.5)	388 (0.4)	14.8 (10.2 to 19.5)	<0.01
	Asian	155 (1.5)	299 (2.3)	1,977 (1.9)	12.0 (10.0 to 14.0)	<0.01
	Black/African American	5,078 (49.2)	6,320 (49.1)	52,496 (49.4)	2.8 (2.5 to 3.2)	<0.01
	Hispanic/Latino^§^	2,454 (23.8)	3,445 (26.7)	26,059 (24.5)	4.5 (4.0 to 5.0)	<0.01
	NH/OPI	10 (0.1)	9 (0.1)	125 (0.1)	—^¶^	—^¶^
	White	2,116 (20.5)	2,355 (18.3)	20,631 (19.4)	1.5 (1.0 to 2.1)	<0.01
	Multiple races	487 (4.7)	388 (3.0)	4,585 (4.3)	-2.5 (-3.6 to -1.4)	<0.01
	Region** of residence at diagnosis					
	Northeast	1,713 (16.6)	1,767 (13.7)	16,326 (15.4)	0.6 (0.0 to 1.2)	<0.01
	Midwest	1,498 (14.5)	1,834 (14.2)	15,821 (14.9)	2.2 (1.6 to 2.8)	<0.01
	South	5,090 (49.3)	6,751 (52.4)	54,283 (51.1)	3.7 (3.4 to 4.1)	<0.01
	West	2,027 (19.6)	2,531 (19.6)	19,828 (18.7)	3.3 (2.8 to 3.9)	<0.01
	Subtotal	10,329 (100.0)	12,883 (100.0)	106,258 (100.0)	2.9 (2.7 to 3.2)	<0.01
30–49 yrs	**Race/Ethnicity**					
	AI/AN	48 (0.4)	64 (0.6)	392 (0.4)	2.9 (-1.0 to 6.9)	0.15
	Asian	298 (2.2)	351 (3.5)	2,807 (2.8)	2.8 (1.3 to 4.3)	<0.01
	Black/African American	3,842 (29.0)	2,855 (28.7)	28,498 (28.3)	-3.6 (-4.1 to -3.2)	<0.01
	Hispanic/Latino^§^	3,218 (24.3)	3,305 (33.2)	28,284 (28.0)	0.6 (0.1 to 1.1)	0.01
	NH/OPI	26 (0.2)	14 (0.1)	179 (0.2)	-2.3 (-7.8 to 3.5)	0.43
	White	5,288 (39.9)	3,113 (31.3)	37,124 (36.8)	-6.4 (-6.7 to -6.0)	<0.01
	Multiple races	548 (4.1)	246 (2.5)	3,578 (3.5)	-8.8 (-10.0 to -7.6)	<0.01
	Region** of residence at diagnosis					
	Northeast	2,178 (16.4)	1,493 (15.0)	16,394 (16.3)	-4.7 (-5.3 to -4.2)	<0.01
	Midwest	1,637 (12.3)	1,177 (11.8)	12,919 (12.8)	-4.0 (-4.7 to -3.4)	<0.01
	South	6,159 (46.4)	4,781 (48.1)	46,485 (46.1)	-3.1 (-3.4 to -2.7)	<0.01
	West	3,294 (24.8)	2,496 (25.1)	25,061 (24.8)	-3.0 (-3.5 to -2.6)	<0.01
	Subtotal	13,268 (100.0)	9,947 (100.0)	100,857 (100.0)	-3.5 (-3.7 to -3.2)	<0.01
≥50 yrs	**Race/Ethnicity**					
	AI/AN	11 (0.3)	13 (0.4)	82 (0.3)	—^¶^	—^¶^
	Asian	45 (1.4)	73 (2.3)	454 (1.6)	7.0 (2.9 to 11.3)	<0.01
	Black/African American	882 (27.4)	762 (23.6)	7,229 (24.9)	-1.8 (-2.9 to -0.8)	<0.01
	Hispanic/Latino^§^	450 (14.0)	625 (19.4)	4,855 (16.7)	4.1 (2.9 to 5.3)	<0.01
	NH/OPI	3 (0.1)	3 (0.1)	34 (0.1)	—^¶^	—^¶^
	White	1,704 (52.9)	1,676 (51.9)	15,461 (53.3)	0.1 (-0.6 to 0.7)	0.87
	Multiple races	125 (3.9)	76 (2.4)	921 (3.2)	-6.7 (-9.1 to -4.2)	<0.01
	Region** of residence at diagnosis					
	Northeast	502 (15.6)	480 (14.9)	4,628 (15.9)	0.2 (-1.1 to 1.4)	0.80
	Midwest	397 (12.3)	472 (14.6)	3,856 (13.3)	2.1 (0.8 to 3.4)	<0.01
	South	1,598 (49.6)	1,466 (45.4)	13,624 (46.9)	-0.6 (-1.3 to 0.1)	0.09
	West	723 (22.5)	810 (25.1)	6,930 (23.9)	0.5 (-0.4 to 1.5)	0.29
	Subtotal	3,220 (100.0)	3,227 (100.0)	29,034 (100.0)	0.1 (-0.3 to 0.6)	0.58
**All ages**	**Total**	**26,816**	**26,057**	**236,150**	**-0.2 (-0.3 to -0.03)**	**0.03**

**Abbreviations:** AIDS = acquired immunodeficiency syndrome; AI/AN = American Indian/Alaska Native; APC = annual percent change; CI = confidence interval; HIV = human immunodeficiency virus; NH/OPI = Native Hawaiian/Other Pacific Islander.

* Data reflect records of all diagnoses of HIV infection, any stage (0, 1, 2, 3 [AIDS], or Unknown) among men who have sex with men. Numbers include diagnoses made from 2008 through 2016 and reported to the national HIV surveillance system by December 31, 2017. Numbers <12 should be interpreted with caution. Data statistically adjusted to account for missing transmission category. Values might not sum to column subtotals and total.

^†^ P<0.05 indicate statistically significant trends.

^§^ Hispanics/Latinos can be of any race.

^¶^ Estimated annual percent change not applicable because of small (value <12) cell sizes.

** Four regions as defined by the U.S. Census comprise: *Region I, Northeast*: Connecticut, Maine, Massachusetts, New Hampshire, New Jersey, New York, Pennsylvania, Rhode Island, and Vermont; *Region II, Midwest*: Illinois, Indiana, Iowa, Kansas, Michigan, Minnesota, Missouri, Nebraska, North Dakota, Ohio, South Dakota, and Wisconsin; *Region III, South*: Alabama, Arkansas, Delaware, District of Columbia, Florida, Georgia, Kentucky, Louisiana, Maryland, Mississippi, North Carolina, Oklahoma, South Carolina, Tennessee, Texas, Virginia, and West Virginia; and *Region IV, West*: Alaska, Arizona, California, Colorado, Hawaii, Idaho, Montana, Nevada, New Mexico, Oregon, Utah, Washington, and Wyoming.

During 2008–2015, the number of MSM living with diagnosed HIV infection increased 4.5% per year, including a 7.7% annual increase among MSM aged 13–29 years, from 40,991 in 2008 to 69,505 in 2015 (Table 2). Among MSM aged 30–49 years, the number living with HIV infection decreased 0.4% per year, from 234,056 in 2008 to 230,003 in 2015. During this period, the number of MSM aged 13–29 years living with HIV increased in 42 jurisdictions, remained stable in five, and decreased in one (APC was not calculated in three jurisdictions, each with cell values <12). The highest APC (11.9%) among MSM in this age group was in Arkansas. 

**TABLE 2:** Trends in number of men who have sex with men* aged ≥13 years living with diagnosed HIV infection, by age group and last known residence at year-end, 2008 and 2015 and estimated annual percent change — National HIV Surveillance System, United States and District of Columbia, 2008–2015

Period/Jurisdiction	Age group (yrs)			
	Total	13–29	30–49	≥50
**Year end 2008**	**No.**	**No. (%)**	**No. (%)**	**No. (%)**
Alabama	4,844	706 (14.6)	2,986 (61.7)	1,152 (23.8)
Alaska	277	14 (5.2)	174 (62.8)	89 (32.0)
Arizona	7,107	657 (9.2)	4,441 (62.5)	2,010 (28.3)
Arkansas	2,247	251 (11.2)	1,438 (64.0)	558 (24.8)
California	69,198	5,351 (7.7)	40,966 (59.2)	22,880 (33.1)
Colorado	6,849	391 (5.7)	3,943 (57.6)	2,515 (36.7)
Connecticut	2,433	214 (8.8)	1,406 (57.8)	814 (33.4)
Delaware	849	117 (13.7)	493 (58.1)	239 (28.1)
District of Columbia	5,427	609 (11.2)	3,199 (58.9)	1,619 (29.8)
Florida	37,098	3,494 (9.4)	23,066 (62.2)	10,538 (28.4)
Georgia	17,290	2,794 (16.2)	11,004 (63.6)	3,491 (20.2)
Hawaii	1,462	64 (4.4)	754 (51.6)	644 (44.1)
Idaho	378	45 (11.8)	229 (60.7)	104 (27.5)
Illinois	15,592	1,900 (12.2)	9,593 (61.5)	4,099 (26.3)
Indiana	4,738	517 (10.9)	3,085 (65.1)	1,135 (24.0)
Iowa	833	70 (8.4)	506 (60.7)	257 (30.9)
Kansas	1,313	163 (12.4)	832 (63.4)	318 (24.2)
Kentucky	2,779	351 (12.6)	1,732 (62.3)	696 (25.0)
Louisiana	6,732	961 (14.3)	4,056 (60.2)	1,715 (25.5)
Maine	624	41 (6.6)	337 (54.0)	246 (39.4)
Maryland	7,168	1,158 (16.2)	4,110 (57.3)	1,899 (26.5)
Massachusetts	6,475	385 (5.9)	3,934 (60.8)	2,156 (33.3)
Michigan	7,218	1,009 (14.0)	4,433 (61.4)	1,776 (24.6)
Minnesota	3,553	294 (8.3)	2,255 (63.5)	1,004 (28.3)
Mississippi	3,382	607 (17.9)	2,078 (61.4)	697 (20.6)
Missouri	6,562	755 (11.5)	4,108 (62.6)	1,698 (25.9)
Montana	172	15 (8.6)	93 (54.1)	64 (37.3)
Nebraska	770	82 (10.6)	507 (65.9)	181 (23.5)
Nevada	3,964	378 (9.5)	2,484 (62.7)	1,101 (27.8)
New Hampshire	510	27 (5.4)	327 (64.1)	156 (30.6)
New Jersey	10,389	1,089 (10.5)	6,299 (60.6)	3,001 (28.9)
New Mexico	1,353	125 (9.3)	810 (59.9)	418 (30.9)
New York	43,998	4,397 (10.0)	26,303 (59.8)	13,298 (30.2)
North Carolina	9,708	1,595 (16.4)	6,090 (62.7)	2,024 (20.8)
North Dakota	98	11 (11.3)	64 (65.0)	23 (23.7)
Ohio	9,633	1,104 (11.5)	5,947 (61.7)	2,583 (26.8)
Oklahoma	2,565	256 (10.0)	1,665 (64.9)	644 (25.1)
Oregon	3,028	219 (7.2)	1,765 (58.3)	1,044 (34.5)
Pennsylvania	9,540	1,147 (12.0)	5,477 (57.4)	2,915 (30.6)
Rhode Island	729	68 (9.3)	429 (58.8)	232 (31.9)
South Carolina	5,605	807 (14.4)	3,529 (63.0)	1,269 (22.6)
South Dakota	154	13 (8.3)	97 (63.2)	44 (28.5)
Tennessee	6,981	970 (13.9)	4,446 (63.7)	1,565 (22.4)
Texas	31,487	3,769 (12.0)	20,068 (63.7)	7,650 (24.3)
Utah	1,270	91 (7.1)	815 (64.2)	364 (28.7)
Vermont	239	14 (5.7)	129 (53.8)	97 (40.5)
Virginia	9,116	1,079 (11.8)	5,540 (60.8)	2,497 (27.4)
Washington	6,311	432 (6.8)	3,859 (61.1)	2,021 (32.0)
West Virginia	755	84 (11.1)	463 (61.3)	208 (27.6)
Wisconsin	2,692	291 (10.8)	1,648 (61.2)	752 (27.9)
Wyoming	97	10 (10.2)	45 (46.8)	42 (42.9)
**Total**	**383,590**	**40,991 (10.7)**	**234,056 (61.0)**	**108,544 (28.3)**
**Year-end 2015**	**Total**	**13–29**	**30–49**	**≥50**
	**No.**	**No. (%)**	**No. (%)**	**No. (%)**
Alabama	6,624	1,351 (20.4)	2,956 (44.6)	2,317 (35.0)
Alaska	330	28 (8.6)	142 (42.9)	160 (48.4)
Arizona	9,868	1,060 (10.7)	4,365 (44.2)	4,443 (45.0)
Arkansas	3,123	509 (16.3)	1,414 (45.3)	1,200 (38.4)
California	87,910	8,035 (9.1)	37,532 (42.7)	42,343 (48.2)
Colorado	7,756	538 (6.9)	2,950 (38.0)	4,269 (55.0)
Connecticut	3,271	380 (11.6)	1,328 (40.6)	1,564 (47.8)
Delaware	1,213	157 (12.9)	493 (40.6)	563 (46.4)
District of Columbia	7,288	822 (11.3)	3,293 (45.2)	3,174 (43.5)
Florida	51,053	5,921 (11.6)	21,302 (41.7)	23,830 (46.7)
Georgia	29,077	5,305 (18.2)	14,380 (49.5)	9,391 (32.3)
Hawaii	1,958	116 (5.9)	656 (33.5)	1,186 (60.6)
Idaho	583	37 (6.3)	240 (41.2)	306 (52.5)
Illinois	21,211	3,258 (15.4)	9,632 (45.4)	8,322 (39.2)
Indiana	6,331	941 (14.9)	2,802 (44.3)	2,588 (40.9)
Iowa	1,419	147 (10.4)	616 (43.4)	656 (46.2)
Kansas	1,710	221 (12.9)	770 (45.0)	719 (42.0)
Kentucky	4,110	584 (14.2)	1,857 (45.2)	1,670 (40.6)
Louisiana	9,397	1,897 (20.2)	4,174 (44.4)	3,326 (35.4)
Maine	904	33 (3.6)	320 (35.3)	552 (61.0)
Maryland	11,631	1,929 (16.6)	5,368 (46.1)	4,335 (37.3)
Massachusetts	8,644	685 (7.9)	3,443 (39.8)	4,517 (52.3)
Michigan	8,922	1,622 (18.2)	3,754 (42.1)	3,546 (39.7)
Minnesota	4,595	470 (10.2)	2,003 (43.6)	2,122 (46.2)
Mississippi	4,668	1,006 (21.6)	2,065 (44.2)	1,597 (34.2)
Missouri	7,899	1,102 (13.9)	3,349 (42.4)	3,448 (43.6)
Montana	333	21 (6.4)	140 (42.2)	171 (51.3)
Nebraska	1,144	137 (12.0)	538 (47.0)	469 (41.0)
Nevada	5,912	753 (12.7)	2,756 (46.6)	2,403 (40.6)
New Hampshire	677	34 (5.0)	271 (40.0)	372 (55.0)
New Jersey	13,050	1,562 (12.0)	5,668 (43.4)	5,820 (44.6)
New Mexico	2,120	222 (10.4)	832 (39.2)	1,067 (50.3)
New York	55,542	6,504 (11.7)	24,833 (44.7)	24,204 (43.6)
North Carolina	14,813	2,614 (17.6)	6,856 (46.3)	5,342 (36.1)
North Dakota	171	22 (12.7)	88 (51.7)	61 (35.6)
Ohio	13,268	2,106 (15.9)	5,577 (42.0)	5,586 (42.1)
Oklahoma	3,531	501 (14.2)	1,571 (44.5)	1,459 (41.3)
Oregon	4,482	314 (7.0)	1,923 (42.9)	2,246 (50.1)
Pennsylvania	13,198	2,120 (16.1)	5,278 (40.0)	5,801 (44.0)
Rhode Island	1,041	98 (9.4)	449 (43.1)	494 (47.5)
South Carolina	7,791	1,460 (18.7)	3,382 (43.4)	2,949 (37.9)
South Dakota	196	13 (6.6)	86 (43.6)	98 (49.8)
Tennessee	8,859	1,471 (16.6)	4,142 (46.8)	3,245 (36.6)
Texas	48,524	8,234 (17.0)	23,116 (47.6)	17,174 (35.4)
Utah	1,614	140 (8.7)	730 (45.2)	744 (46.1)
Vermont	397	16 (4.0)	144 (36.3)	238 (59.8)
Virginia	11,500	1,794 (15.6)	4,767 (41.5)	4,939 (43.0)
Washington	8,287	621 (7.5)	3,649 (44.0)	4,017 (48.5)
West Virginia	975	85 (8.7)	414 (42.4)	477 (48.9)
Wisconsin	3,644	506 (13.9)	1,523 (41.8)	1,616 (44.3)
Wyoming	151	8 (5.0)	68 (45.3)	75 (49.7)
**Total**	**522,718**	**69,505 (13.3)**	**230,003 (44.0)**	**223,210 (42.7)**
**2008–2015**	**Total**	**13–29**	**30–49**	**≥50**
	**APC (95% CI)**	**APC (95% CI)**	**APC (95% CI)**	**APC (95% CI)**
Alabama	4.8 (4.4 to 5.3)	9.5 (8.5 to 10.6)	0.2 (-0.4 to 0.8)	10.5 (9.7 to 11.4)
Alaska	2.0 (0.2 to 3.7)	10.9 (3.9 to 18.4)	-3.4 (-5.6 to -1.1)	7.8 (4.8 to 10.8)
Arizona	4.7 (4.4 to 5.0)	7.4 (6.2 to 8.5)	-0.6 (-1.1 to -0.1)	12.0 (11.3 to 12.6)
Arkansas	4.9 (4.3 to 5.5)	11.9 (10.1 to 13.7)	-0.4 (-1.2 to 0.4)	11.6 (10.4 to 12.8)
California	3.4 (3.3 to 3.5)	5.9 (5.5 to 6.3)	-1.4 (-1.6 to -1.2)	9.2 (9.0 to 9.4)
Colorado	1.8 (1.5 to 2.2)	4.4 (3.0 to 5.9)	-4.3 (-4.8 to -3.8)	8.0 (7.4 to 8.5)
Connecticut	4.1 (3.6 to 4.7)	8.0 (6.1 to 10.0)	-1.0 (-1.8 to -0.2)	9.5 (8.5 to 10.5)
Delaware	5.1 (4.1 to 6.1)	4.0 (1.5 to 6.7)	-0.4 (-1.8 to 1.0)	13.2 (11.4 to 14.9)
District of Columbia	4.2 (3.9 to 4.6)	4.6 (3.4 to 5.7)	0.5 (-0.1 to 1.0)	9.8 (9.1 to 10.5)
Florida	4.7 (4.6 to 4.9)	7.7 (7.2 to 8.2)	-1.2 (-1.4 to -1.0)	12.5 (12.2 to 12.8)
Georgia	7.5 (7.3 to 7.7)	9.0 (8.5 to 9.6)	3.7 (-3.4 to 4.0)	14.8 (14.3 to 15.3)
Hawaii	3.9 (3.1 to 4.6)	7.1 (3.7 to 10.6)	-2.6 (-3.7 to -1.5)	9.0 (7.9 to 10.1)
Idaho	6.1 (4.6 to 7.6)	-2.8 (-7.1 to 1.7)	0.3 (-1.6 to 2.3)	16.0 (13.5 to 18.6)
Illinois	4.4 (4.2 to 4.7)	7.7 (7.1 to 8.4)	0.0 (-0.3 to 0.3)	10.7 (10.3 to 11.2)
Indiana	4.2 (3.8 to 4.6)	9.3 (8.0 to 10.5)	-1.6 (-2.1 to -1.0)	12.3 (11.5 to 13.1)
Iowa	7.6 (6.7 to 8.6)	10.5 (7.3 to 13.8)	2.9 (1.6 to 4.2)	13.7 (12.0 to 15.4)
Kansas	3.8 (3.0 to 4.6)	3.9 (1.7 to 6.2)	-1.1 (-2.1 to -0.002)	11.9 (10.4 to 13.5)
Kentucky	5.8 (5.3 to 6.4)	7.7 (6.2 to 9.2)	0.9 (0.2 to 1.6)	13.5 (12.5 to 14.6)
Louisiana	4.9 (4.6 to 5.3)	10.1 (9.2 to 11.0)	0.3 (-0.2 to 0.8)	9.9 (9.2 to 10.7)
Maine	5.1 (4.0 to 6.3)	-5.7 (-10.7 to -0.5)	-0.6 (-2.2 to 1.0)	11.7 (10.0 to 13.4)
Maryland	6.8 (6.4 to 7.1)	6.7 (5.9 to 7.5)	3.4 (2.9 to 3.8)	12.4 (11.7 to 13.1)
Massachusetts	4.4 (4.0 to 4.8)	9.3 (7.8 to 10.8)	-1.8 (-2.3 to -1.3)	11.2 (10.6 to 11.8)
Michigan	3.2 (2.9 to 3.6)	7.2 (6.4 to 8.1)	-2.3 (-2.8 to -1.9)	10.4 (9.7 to 11.0)
Minnesota	3.7 (3.2 to 4.2)	5.6 (3.9 to 7.2)	-1.8 (-2.5 to -1.1)	11.4 (10.5 to 12.3)
Mississippi	4.7 (4.2 to 5.2)	7.4 (6.2 to 8.6)	-0.2 (-0.9 to 0.5)	12.5 (11.4 to 13.6)
Missouri	2.7 (2.4 to 3.1)	5.2 (4.2 to 6.3)	-2.9 (-3.4 to -2.4)	10.9 (10.2 to 11.6)
Montana	8.9 (6.9 to 10.9)	1.0 (-5.7 to 8.3)	5.5 (2.6 to 8.4)	14.2 (11.0 to 17.6)
Nebraska	5.5 (4.4 to 6.5)	7.4 (4.2 to 10.7)	0.5 (-0.8 to 1.9)	14.0 (12.0 to 16.0)
Nevada	5.7 (5.3 to 6.2)	10.9 (9.4 to 12.3)	1.1 (0.5 to 1.7)	11.7 (10.8 to 12.5)
New Hampshire	4.2 (2.9 to 5.5)	2.9 (-2.4 to 8.4)	-2.7 (-4.4 to -0.9)	13.1 (10.9 to 15.3)
New Jersey	3.4 (3.1 to 3.7)	5.3 (4.4 to 6.2)	-1.6 (-2.0 to -1.1)	10.1 (9.5 to 10.6)
New Mexico	5.8 (5.1 to 6.6)	7.9 (5.3 to 10.5)	-0.3 (-1.3 to 0.7)	13.1 (11.8 to 14.4)
New York	3.4 (3.3 to 3.6)	5.9 (5.4 to 6.3)	-0.9 (-1.1 to -0.7)	8.9 (8.7 to 9.2)
North Carolina	6.1 (5.8 to 6.4)	7.1 (6.3 to 7.8)	1.6 (1.2 to 1.9)	14.6 (13.9 to 15.2)
North Dakota	8.8 (5.8 to 11.9)	—^†^	4.6 (0.9 to 8.4)	15.6 (9.8 to 21.8)
Ohio	4.6 (4.3 to 4.9)	9.6 (8.7 to 10.4)	-1.0 (-1.4 to -0.6)	11.4 (10.9 to 12.0)
Oklahoma	4.6 (4.0 to 5.2)	9.8 (8.0 to 11.5)	-1.1 (-1.8 to -0.3)	12.5 (11.4 to 13.6)
Oregon	5.3 (4.8 to 5.8)	4.4 (2.5 to 6.3)	0.7 (-0.02 to 1.4)	11.2 (10.4 to 12.0)
Pennsylvania	4.6 (4.3 to 4.9)	8.5 (7.7 to 9.3)	-0.7 (-1.1 to -0.3)	10.3 (9.8 to 10.8)
Rhode Island	4.9 (3.9 to 6.0)	5.0 (1.6 to 8.4)	0.4 (-1.0 to 1.9)	11.3 (9.5 to 13.2)
South Carolina	4.6 (4.2 to 5.0)	8.9 (7.9 to 9.9)	-0.9 (-1.4 to -0.4)	12.3 (11.5 to 13.1)
South Dakota	3.5 (1.1 to 6.0)	—^†^	-2.4 (-5.6 to 0.8)	13.1 (8.8 to 17.5)
Tennessee	3.4 (3.0 to 3.7)	5.9 (5.0 to 6.8)	-1.1 (-1.5 to -0.6)	10.8 (10.1 to 11.5)
Texas	6.4 (6.2 to 6.5)	11.6 (11.2 to 12.1)	1.9 (1.7 to 2.1)	12.2 (11.9 to 12.5)
Utah	3.6 (2.8 to 4.5)	6.0 (2.9 to 9.1)	-1.6 (-2.7 to -0.5)	10.5 (9.0 to 11.9)
Vermont	8.6 (6.8 to 10.6)	5.5 (-2.5 to 14.1)	2.3 (-0.2 to 5.0)	15.2 (12.3 to 18.2)
Virginia	3.4 (3.1 to 3.7)	7.2 (6.3 to 8.1)	-2.0 (-2.5 to -1.6)	10.2 (9.6 to 10.7)
Washington	3.9 (3.6 to 4.3)	4.4 (3.0 to 5.8)	-0.9 (-1.4 to -0.4)	10.4 (9.7 to 11.0)
West Virginia	3.7 (2.7 to 4.8)	1.5 (-1.9 to 5.0)	-1.6 (-3.0 to -0.2)	11.9 (10.1 to 13.8)
Wisconsin	4.6 (4.0 to 5.2)	7.9 (6.3 to 9.6)	-1.1 (-1.9 to -0.3)	11.7 (10.7 to 12.7)
Wyoming	5.9 (3.0 to 8.9)	—^†^	4.9 (0.9 to 9.2)	8.5 (4.2 to 13.1)
**Total**	**4.5 (4.4 to 4.5)**	**7.7 (7.5 to 7.8)**	**-0.4 (-0.4 to -0.3)**	**10.8 (10.7 to 10.9)**

**Abbreviations:** AIDS = acquired immunodeficiency syndrome; APC = annual percent change; CI = confidence interval; HIV = human immunodeficiency virus.

* Data reflect records of all diagnoses of HIV infection, any stage (0, 1, 2, 3 [AIDS], or Unknown) among men who have sex with men. Numbers include cases diagnosed through 2015 and reported to the national HIV surveillance system by December 31, 2017. Numbers <12 should be interpreted with caution. Data statistically adjusted to account for missing transmission category. Values might not sum to column totals.

^†^ Estimated annual percent change not applicable because of small (value <12) cell sizes.

The number of MSM aged ≥50 years living with HIV infection increased in all jurisdictions, ranging from an estimated average of 7.8% in Alaska to 16.0% per year in Idaho. Among MSM aged ≥50 years, the number of persons living with HIV infection increased 10.8% per year, from 108,544 in 2008 to 223,210 in 2015. In 12 jurisdictions, at least half of MSM living with diagnosed HIV infection were aged ≥50 years. Seven of these states were in the West (Colorado, Hawaii, Idaho, Montana, New Mexico, Oregon, and Wyoming), four were in the Northeast (Maine, New Hampshire, Massachusetts, and Vermont) and one was in the Midwest (South Dakota). Nine of 10 states with the highest percentages of MSM living with diagnosed HIV infection aged 13–29 years were in the South.

## Discussion

During 2008–2016, the annual number of diagnoses of HIV infection among MSM increased 3% per year among persons aged 13–29 years, decreased 4% per year among those aged 30–49 years and was stable among those aged ≥50 years. The number of diagnoses among MSM aged 13–29 years was four times that among MSM ≥50 years.

Racial/ethnic disparities in the occurrence of annual diagnoses of HIV infection persisted, particularly among younger MSM. Compared with non-Hispanic whites, blacks and Hispanics/Latinos accounted for a disproportionate number of cases. Among MSM aged 13–29 years, American Indians/Alaska Natives, Asians, and residents of the South experienced the steepest increases in trends in annual diagnoses of HIV infection compared with other racial/ethnic groups and other U.S. regions; however, the numbers of annual diagnoses of HIV infection among American Indian/Alaska Native and Asian MSM were small.

During 2008–2015, the number of MSM aged ≥50 years living with diagnosed HIV infection increased by 11% per year, and at year-end 2015, this group accounted for the largest age group of MSM living with diagnosed HIV infection, presumably as a result of increased survival associated with widespread use of antiretroviral therapy (*7*), surviving middle age, and advancing to the older group. In light of the large and increasing percentage of older MSM living with diagnosed HIV infection, care and treatment that includes achieving viral suppression and managing age-related comorbidities is essential (*8*).

The increase in annual diagnosis of HIV infections among younger MSM might reflect increased HIV testing, in addition to ongoing transmission. Intensified efforts to increase the rate of HIV testing are particularly important for younger MSM because they account for the highest percentage of MSM with undiagnosed HIV infection (*9*). Increasing HIV testing can help diagnose HIV infection sooner, enable MSM to access HIV treatment (*4*), and reduce HIV transmission to others (*10*). To avert the largest number of infections and improve health outcomes, MSM should be tested at least once a year (*3*) and, if positive, linked to and retained in HIV medical care to achieve viral suppression (*4*). Those testing negative should receive HIV prevention services, including PrEP (*5*).

The findings in this report are subject to at least three limitations. First, some cases of HIV infection are reported to CDC without an identified risk factor. Statistical adjustments were applied for missing risk factor information (*6*); as a result of this imputation, estimated numbers of reported cases attributable to male-to-male sexual contact are higher than numbers of cases reported to CDC with male-to-male sexual contact indicated. Second, although NHSS data reflect high completeness of reporting from jurisdictions,^§^ some diagnoses of HIV infection might not have been reported to CDC (resulting in an underestimation), and some might reflect duplicate reporting (resulting in an overestimation). These are mitigated by collecting all HIV-related laboratory and case information from providers of surveillance data and intrastate and interstate deduplication,^¶^ yielding reliable numbers of annual diagnoses. Finally, because of small numbers of annual HIV diagnoses in American Indians/Alaska Natives and Asians, comparisons of trends by race/ethnicity should be undertaken with caution.

These findings highlight the need to strengthen interventions for all MSM, including risk-reduction counseling and screening, and provision of PrEP to MSM at high risk for HIV acquisition (*5*). Promotion of care and treatment by public health agencies and private sector partners to achieve viral suppression among MSM with diagnosed HIV infection will improve health outcomes and reduce transmission to others, particularly if prevention efforts are tailored to specific age groups. To reduce disparities in HIV transmission and acquisition, more widespread implementation of interventions** for those with disproportionate risk and burden of HIV infection, such as black and Hispanic/Latino MSM, are needed.

**Summary**

**What is already known about this topic?**

In 2016, 67% of diagnosed human immunodeficiency virus (HIV) infections were attributed to male-to-male sexual contact.

**What is added by this report?**

During 2008–2016, the number of HIV diagnoses increased 3% annually among men who have sex with men (MSM) aged 13–29 years. The number of HIV diagnoses among MSM aged 13–29 years was four times that of MSM aged ≥50 years. Racial/ethnic inequities in HIV persisted, particularly among younger black/African American and Hispanic/Latino MSM.

**What are the implications for public health practice?**

MSM may be tested at least annually and, if positive, linked to and retained in HIV medical care. Those testing negative might benefit from prevention services, including preexposure prophylaxis. Strengthened efforts can reduce racial/ethnic inequities.

## Footnotes

* Excluding men who have sex with men and inject drugs.

† The National HIV Surveillance System is the primary source for monitoring HIV trends in the United States. Through the system, information about cases of HIV infection is collected, analyzed, and disseminated.

§ CDC. Evaluation Framework. Oral presentation at the PS18-1802: Integrated HIV Surveillance and Prevention Programs for Health Departments: Recipient Orientation Meeting. Jun 6, 2018. Atlanta, Georgia.

¶ Mitsch A, Tang T, Whitmore S. Accurate monitoring of HIV in the United States—CDC's Routine Interstate Duplicate Review 2005–2008. 19th International AIDS Conference, July 22–27, 2012, Washington DC, USA. https://www.researchgate.net/publication/272827093_Accurate_monitoring_of_HIV_in_the_United_States_-_CDC%27s_Routine_Interstate_Duplicate_Review_2005-2008.

** https://www.cdc.gov/msmhealth/msm-programs.htm.
